# The mechanism and mitigation of niacin-induced flushing

**DOI:** 10.1111/j.1742-1241.2009.02099.x

**Published:** 2009-09

**Authors:** V S Kamanna, S H Ganji, M L Kashyap

**Affiliations:** Department of Veterans Affairs Healthcare System, Atherosclerosis Research Center, CA, USA, and the Department of Medicine, University of California–IrvineCA, USA

## Abstract

**Aims:**

To summarise the metabolic responses to niacin that can lead to flushing and to critically evaluate flushing mitigation research.

**Methods and results:**

This comprehensive review of the mechanism of action of niacin-induced flushing critically evaluates research regarding flushing mitigating formulations and agents. Niacin induces flushing through dermal Langerhans cells where the activation of G protein-coupled receptor 109A (GPR109A) increases arachidonic acid and prostaglandins, such as prostaglandin D_2_ (PGD_2_) and prostaglandin E_2_ (PGE_2_), subsequently activating prostaglandin D_2_ receptor (DP_1_), prostaglandin E_2_ receptor (EP_2_) and prostaglandin E receptor 4 (EP_4_) in capillaries and causing cutaneous vasodilatation. Controlling niacin absorption rates, inhibiting prostaglandin production, or blocking DP_1_, EP_2_ and EP_4_ receptors can inhibit flushing. Niacin extended-release (NER) formulations have reduced flushing incidence, duration and severity relative to crystalline immediate-release niacin with similar lipid efficacy. Non-steroidal anti-inflammatory drugs (NSAIDs), notably aspirin given 30 min before NER at bedtime, further reduce flushing. An antagonist to the DP_1_ receptor (laropiprant) combined with an ER niacin formulation can reduce flushing; however, significant residual flushing occurs with clinically-relevant dosages.

**Conclusions:**

Niacin is an attractive option for treating dyslipidemic patients, and tolerance to niacin-induced flushing develops rapidly. Healthcare professionals should particularly address flushing during niacin dose titration.

**Review Criteria**

Research regarding the mechanism of action of niacin and the formulations and agents used in the mitigation of flushing were systematically reviewed and summarised. PubMed was searched from 1960 to 2008 using the terms niacin, flushing, laropiprant, prostaglandins and aspirin. All hits were reviewed for inclusion of mechanism of action, and pertinent articles were included, excluding results which had been subsequently disproven.

**Message for the Clinic**

Niacin, an attractive option for treating dyslipidaemic patients, substantially improves most lipid parameters associated with atherosclerosis. However, flushing is a common non-allergenic response to niacin that reduces medication compliance. Several options to mitigate flushing symptoms prior to the development of tolerance are discussed. Clinical trials and practical experience indicate that a high level of medication compliance can be achieved if healthcare providers counsel their patients prior to starting niacin therapy.

## Introduction

Niacin, either alone or in combination with a statin, safely and effectively addresses most lipid abnormalities in patients with mixed dyslipidaemia. Therapeutically used for more than 50 years, niacin is the most effective clinically available agent for increasing high-density lipoprotein cholesterol (HDL-C) levels. In most patients, niacin increases HDL-C by 20–40% (1–5). Niacin also has beneficial effects on all known pro-atherogenic lipid parameters, including lowering low-density lipoprotein cholesterol (LDL-C), non-HDL-C and triglycerides. It is the only current lipid therapy that decreases Lp(a), an independent risk factor for atherosclerosis (2,6). Niacin also has favourable effects on lipid particle size; it reduces small, dense LDL (7) while increasing cardio-protective HDL, as measured by either particle size (HDL_2_) (8) or by apolipoprotein profile (HDL containing apolipoprotein A-I without apolipoprotein A-II) (9). These alterations in lipids are clinically meaningful, as treatment with niacin has been associated with significant reductions in cardiovascular events and morbidity (10) and, in combination with statins, with regression of atherosclerotic cardiovascular disease (11).

Despite niacin’s numerous beneficial lipid effects, patient compliance to long-term therapy is challenged by flushing, a common side effect of niacin. A significant portion of the effects of niacin on flushing results from activation of the niacin receptor G protein-coupled receptor 109A (GPR109A) in dermal Langerhans cells (12,13), leading to the production of prostaglandins, including prostaglandin D_2_ (PGD_2_) and prostaglandin E_2_ (PGE_2_), which act on receptors in the capillaries. Flushing is characterised by cutaneous vasodilatation and manifests itself as redness or warmth of the skin, sometimes accompanied by tingling or itching. The onset of flushing can occur rapidly and usually lasts about 1 h. It is a transient, non-allergic response, but it may result in patient discomfort. In a randomised dose escalation trial, the mean incidence of flushing episodes decreased from the highest (2.7 per patient per month) with a 500-mg dose, and decreased to 1.1 with a 2000-mg dose (14). The incidence of flushing decreases with time as quickly as 1 week (15), because tolerance develops via decreased prostanoid (PGD2, a major mediator of flushing) secretion with repeated doses of niacin (15). This review summarises the metabolic responses to niacin that can lead to flushing and examines the current strategies to manage the effects of flushing in patients.

## Niacin: mechanism of action

Physiologically, niacin influences lipoprotein metabolism by decreasing triglyceride synthesis via multiple pathways. In adipocytes, it inhibits the lipolysis of triglycerides and retards the mobilisation of free fatty acids (FFAs) to the plasma. As the liver uses plasma FFAs as substrates to form triglycerides, hepatic triglyceride production is decreased. Niacin can also reduce *de novo* synthesis of triglycerides in the liver by inhibiting the enzyme that catalyses the terminal reaction in cellular triglyceride synthesis, diacylglycerol acyltransferase 2 (DGAT2) (16). A reduction in hepatic triglyceride synthesis has important downstream effects on other lipoproteins. The production of very low density lipoprotein (VLDL) particles and thus VLDL-C is dependent on triglyceride synthesis in the liver, and IDL-C and LDL-C are derived from VLDL-C. Therefore, by decreasing hepatic triglyceride synthesis, niacin impairs synthesis of VLDL and thus decreases circulating levels of VLDL-C and subsequently IDL-C and LDL-C.

The mechanism through which niacin increases HDL-C is under investigation. Niacin does not appear to directly increase hepatic HDL particle or apolipoprotein A-I (the most abundant lipoprotein in HDL) synthesis. Instead, niacin likely prevents the catabolism of circulating HDL through several ways. Niacin decreases HDL catabolism by decreasing the fractional clearance of ApoA-I associated with HDL (17). When liver cells were treated with niacin, their uptake of HDL was inhibited, but the uptake of its cholesteryl ester was not (9). By preventing the hepatic catabolism of HDL, but not the uptake of the cholesteryl ester, niacin can increase the amount of circulating functional HDL, and thus facilitate reverse cholesterol transport. Therefore, the amount of HDL-ApoA-I-containing lipoprotein particles would be increased without increasing the rate of production of these particles. Recent research indicates that niacin decreases hepatocyte surface expression of beta-chain adenosine triphosphate (ATP) synthase (18), a mitochondrial protein reported to mediate hepatic HDL holoparticle endocytosis (19). These findings suggest that niacin, by downregulating hepatocyte surface expression of the beta-chain ATP synthase, reduces hepatic removal of HDL through holoparticle endocytosis, thus implicating a potential cellular receptor site for niacin’s action to raise plasma HDL.

Some of niacin’s effects on FFAs may be because of its properties as a high-affinity agonist for the G-protein coupled receptor, GPR109A protein-upregulated in macrophages by interferon-γ (PUMA-G) in mice (20–22). In humans, GPR109A is expressed in adipocytes, dermal immune cells (Langerhans) and macrophages, but not in the liver (23). Mice that lack the PUMA-G GPR109A receptor do not show improvements in lipid parameters after niacin treatment (21). Whether this mechanism operates in humans is unclear because of rebound lipolysis that occurs after the acute initial reduction (24). There is strong evidence that activation of the GPR109A (12) receptor in Langerhans cells (13) leads to flushing, even though the antilipolytic effects of niacin are likely mediated through GPR109A receptors in the adipocytes. Mice that lack the PUMA-G GPR109A receptor do not flush when administered niacin, but can flush after the receptor is restored in immune cells following a bone marrow transplant from normal mice (12).

In addition to lipid effects, recent research also suggested that niacin beneficially affects vascular inflammatory processes involved in atherogenesis. The findings from these studies indicate for the first time that niacin inhibits vascular inflammation by decreasing endothelial reactive oxygen species (ROS) production resulting in decreased endothelial expression of redox-sensitive genes, vascular cell adhesion molecule-1 (VCAM-1) and monocyte chemotactic protein-1 (MCP-1), and monocyte/macrophage adhesion and accumulation, key events in early atherogenesis (25). These *in vitro* studies describe a novel mechanistic role for niacin in decreasing atherosclerosis beyond its conventional role as a lipid-regulating agent.

## Niacin-induced flushing: basic mechanism and mediators

Flushing symptoms occur following vasodilatation of small capillaries under the skin, a response that can be mediated via histamine/bradykinin or prostaglandins. Flushing is not unique to niacin; it has also been reported frequently by patients taking phosphodiesterase inhibitors, selective serotonin reuptake inhibitors (SSRIs), selective oestrogen receptor modulators (SERMs), adenosine and tretinoin. Topical and oral administration of niacin has not been associated with increases in blood levels of either histamine or bradykinin, suggesting that niacin-induced flushing is not mediated by mast cells (26,27). The release of histamine or bradykinin causes a substantial rise in nitric oxide, which leads to increased intracellular release of cyclic guanosine monophosphate (cGMP) and vasodilatation. Elimination of endothelial nitric oxide synthase (eNOS), an enzyme critical for NO production, did not stop niacin-induced flushing in mice (12), providing further support that the histamine/bradykinin pathway is not involved in niacin-induced flushing.

Prostaglandins (PGs), specifically forms D_2_ and E_2_, have been identified as participants in the niacin-induced flushing response (28,29). PGs, prostacylcins, thromboxanes and leukotrienes, collectively considered eicosanoids, are hormone-like chemical messengers derived from arachidonic acid. PGs have numerous biological effects, including essential roles in platelet aggregation, neurotransmitter release, and inflammatory and vasomotor responses. Individual prostaglandins can have both positive and negative effects (i.e. pro-inflammatory or anti-inflammatory, vasodilatory or vasoconstrictive) depending upon their concentration, relative proportion to other prostaglandins and expression of receptor types. As prostaglandins are rapidly metabolised and have short half-lives, their metabolic effects are typically localised and can be variable in different parts of the body.

## The arachidonic acid cascade

**Figure 1:**
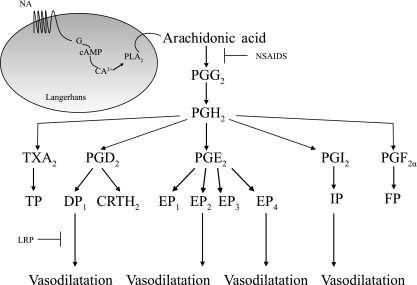
Niacin activates the arachidonic acid cascade to induce vasodilatation. Niacin activates the G-protein coupled receptor 109A (GPR109A) to increase cAMP and releases arachidonic acid from cell membranes. Arachidonic acid is metabolised to produce prostaglandins, prostacyclin and thromboxane. Activation of the prostaglandin D_2_ receptor (DP_1_), prostaglandin E_2_ receptor (EP_2_), EP4 and IP receptors can lead to vasodilatation that may contribute to flushing. NSAIDs block the metabolism of arachidonic acid, while LRP blocks DP_1_-mediated vasodilatation. cAMP, cyclic AMP; PLA2, phospholipase A2; PG, Prostaglandin; CRTH2, chemoattractant receptor homologous-molecule expressed on T helper type 2; NA, nicotinic acid; NSAIDs, non-steroidal anti-inflammatory drugs; LRP, laropiprant

Activation of the GPR109A receptor by niacin initiates a signalling cascade that ultimately results in the production of prostaglandins, and thus, flushing (Figure 1). In Langerhans cells, niacin can activate GPR109A to increase intracellular Ca^2+^ (13). This Ca^2+^ increase triggers phospholipases, predominantly Phospholipase A_2_ (PLA_2_), to release arachidonic acid from cellular lipid stores (30). Free arachidonic acid serves as a precursor to the production of eicosanoids, including lipoxygenases, thromboxanes and prostaglandins.

The production of prostaglandins from arachidonic acid involves a complex cascade of enzymes. The first step is the metabolism of arachidonic acid to prostaglandin H_2_ (PGH_2_) by PGH synthase, an enzyme that has both cyclooxygenase and endoperoxidase activity, but is commonly referred to as COX. Sequential metabolism of arachidonic acid by COX produces prostaglandin G_2_ (PGG_2_), which is then reduced to PGH_2_, an unstable intermediate. Aspirin and related non-steroidal anti-inflammatory drugs (NSAIDs) can prevent the synthesis of prostaglandins by inhibiting both isoforms of COX (COX-1 and COX-2). The inhibition of COX also eliminates the flushing response to niacin (28,31–33). From PGH_2_, multiple prostaglandin synthase enzymes synthesise PGD_2_, PGE_2_, prostaglandin I_2_ (PGI_2_, prostacyclin), thromboxane A_2_ (TXA_2_, thromboxane) and prostaglandin F_2α_ (PGF_2α_).

After their synthesis, prostaglandins exert their effects locally through downstream receptors. There are currently five known prostaglandin receptor families activated by prostaglandins: DP, prostaglandin E receptor (EP), prostacyclin receptor (IP), thromboxane A2 receptor (TP) and F2α receptor (FP). The EP receptors are further divided into four subtypes, prostaglandin E_1_ receptor (EP_1_), EP_2_, EP_3_ and EP_4_, and there are two DP receptor subtypes, DP_1_ and DP_2_, also called chemoattractant receptor homologous-molecule expressed on T helper type 2 (CRTH2). The receptors are categorised by their affinity for each respective prostaglandin agonist. PGE_2_ binds to the EP family, PGD_2_ binds to DP, PGI_2_ binds to IP and so forth. The downstream effects of the activation of each individual receptor can be dependent on the tissue expression of the receptor as well as the G-protein to which the receptor is coupled. These conditions allow some of the receptors to have opposing actions in the same tissue and result in complicated predictions of receptor activation. Broadly, these receptors can be divided into two groups, ‘relaxant’ or ‘excitatory’, according to their effects on smooth muscle. The relaxant receptors consist of DP, EP_2_, EP_4_ and IP, whereas TP, EP_1_ and FP are categorised as excitatory (34). Based on this classification, the relaxant receptors would be expected to play a role in cutaneous vasodilatation, while the excitatory receptors would act as vasoconstrictors.

## Vasodilatory PGs

Through their downstream receptors, PGD_2_, PGE_2_ and PGI_2_ can all exhibit vasodilatory effects on smooth muscle cells in the vasculature, among other effects. Through the DP_1_ receptor, PGD_2_ inhibits platelet aggregation and mediates smooth muscle relaxation/contraction. Although PGD_2_ is known to have vasodilatory properties in the vascular endothelium, it can behave as a vasoconstrictor at higher concentrations and in separate tissues (35). PGD_2_ can also act through the chemoattractant CRTH2 (DP_2_) receptor, whose biological role appears to be regulating inflammatory allergic and asthmatic responses (36). PGE_2_ is perhaps the most widely produced prostaglandin, and as the highest-affinity agonist for the EP receptor family, it exerts the most diverse and versatile effects (37). The EP_2_ receptors are localised to smooth muscle in the trachea, GI tract and vascular system. They, along with EP_4_ receptors, are relaxant receptors and induce vasodilatation of various blood vessels through increasing cAMP. The PGE_2_-EP_4_ receptor pathway may also mediate some anti-inflammatory effects and facilitate mobilisation, migration and maturation of Langerhans cells in the skin (38). Prostacyclin is the major arachidonic acid product in vascular tissues (39). PGI_2_ is produced in blood vessels where it is a potent vasodilator and inhibitor of platelet aggregation through the IP receptor (34).

## Vasoconstrictory PGs

Thromboxane and PGF_2α_, acting through the TP and FP receptors, respectively, are both potent vasoconstrictors. Thromboxane A2 (TXA_2_) plays an extensive role in haemodynamics and cardiovascular function. The majority of thromboxane produced *in vivo* is made by platelets, where it exhibits opposing actions to PGI_2_. TXA_2_ can increase platelet aggregation, and thus deficiencies in the TP receptor can lead to bleeding disorders. TP receptors are expressed in the thymus, spleen and lungs. Increased TXA_2_ has been linked to cardiovascular diseases including acute myocardial ischaemia and heart failure (40). In humans, PGF_2α_ is a potent constrictor of pulmonary arteries and veins (41,42). It increases blood pressure in experimental animals, but not humans. As PGF_2α_ and TXA_2_ are potent vasoconstrictors, they are unlikely candidates to induce flushing, but may blunt the actions of vasodilatory prostaglandins.

## Prostaglandins involved in flushing

Several prostaglandins with vasodilatory properties are influenced by niacin. Specifically, levels of PGD_2_, PGE_2_, PGI_2_ and their metabolites have been shown to be increased as quickly as 12–45 min after niacin treatment (15,26,32,43). Their respective receptors, DP, EP_2_ and EP_4_ and IP, can all induce relaxation of blood vessels. After oral niacin treatment, PGD_2_ levels in the venous blood draining the skin are 14–1200 times higher than the levels in arterial blood (44). The production of PGI_2_ and PGD_2_ decreases after repetitive administration of niacin in parallel with the development of flushing tolerance (15). Further, methylnicotinate, which can deliver niacin transdermally, applied to a subject’s arm releases PGD_2_ only in the exposed arm, with no change in the untreated arm (44). Langerhans cells express PGD_2_ and PGE_2_ synthase enzymes (13), indicating they can produce PGD_2_ and PGE_2_ to activate receptors on blood vessels that lead to vasodilatation and contribute to flushing side effects. PUMA-G-deficient mice still flush when administered PGD_2_ (12). Likewise, humans pretreated with the NSAID indomethacin still flush when challenged with PGE (28). Separate deletions of the DP_1_, EP_2_ and EP_4_ in mice result in 40%, 20% and 40% reductions in flushing response, respectively, relative to normal mice after niacin administration (12). In comparison, deletions of COX in mice completely eliminate flushing after niacin treatment (12). Mice that do not express the IP receptor still flush after niacin (12). These experiments indicate that PGD_2_ and PGE_2_, signalling through the DP_1_, EP_2_ and EP_4_ receptors, are likely responsible for the flushing side effects of niacin.

## Other products of arachidonic acid

Besides COX, arachidonic acid can be metabolised by a family of enzymes called lipoxygenases to produce leukotrienes (LT), a group of inflammatory lipid mediators. They are released from neutrophils, eosinophils, mast cells and macrophages to play a role in innate immunity (45). Leukotrienes mediate asthma effects, mucus secretion and bronchoconstriction/bronchodilation. Leukotriene B_4_ (LTB_4_) applied directly to the skin can cause vasodilatation that is not decreased by COX inhibitors, indicating that the vasodilatation is not mediated by prostaglandins (46). The downstream mechanism of LTB_4_-mediated vasodilatation is currently unknown. Patients treated with niacin have shown evidence of increases in leukotriene E4 (LTE_4_) but not LTB_4_ (47). Any potential effects of LTE_4_ on niacin-induced flushing have not been reported, and its role in vasodilatation is unclear.

## Managing patient flushing

Considering niacin’s beneficial effects on nearly all lipid parameters associated with cardiovascular risk, and recent demonstration of its vascular anti-inflammatory properties (25), strategies to minimise or eliminate flushing should be deemed important to increase patient compliance to niacin therapy. As attenuation to flushing rapidly develops (28), dose titration is important in reducing flushing in patients. As a result of niacin’s mechanism of action, flushing can be managed in three ways: (i) by controlling the absorption rate of niacin; (ii) by preventing the production of prostaglandins; or (iii) by simultaneously blocking the DP_1_, EP_2_ and EP_4_ receptors. Educating patients about the benefits of niacin therapy may also increase the likelihood that patients are willing to tolerate any of the minor bothersome effects of flushing (48).

## New formulations of niacin

Flushing can persist as long as plasma niacin levels are rising, but abates when constant plasma niacin levels are reached (49). Therefore, flushing is also related to the rate of niacin absorption, as a higher rate of absorption is associated with a higher rate of flushing (50,51). Crystalline, immediate-release (IR) niacin is rapidly absorbed by the body, and peak blood levels can be reached in as quickly as 30–60 min (52). As such, flushing incidence among patients taking IR niacin is close to 100%, and flushing was the major reason for discontinuation of IR niacin in several studies (53–55). To reduce flushing, several alternate formulations of niacin have been made. Sustained-release (SR) niacin formulations were created to delay niacin absorption during treatment. Although SR niacin decreases flushing, it can also cause hepatotoxicity and has shown inconsistent effects on lipids (55,56). Inositol hexanicotinate is commonly referred to as no-flush niacin or flush-free niacin, but this dietary supplement has not been shown to have any beneficial effects on lipid parameters (57,58).

Newer prescription niacin extended-release (NER) formulations (Niaspan®, Abbott, Abbott Park, IL, USA) have shown reduced flushing side effects relative to IR niacin while having equivalent efficacy to alter lipid parameters (5). Among healthy subjects receiving a single 2000 mg dose of a coated formulation of NER, there was a 42% reduction in median flush intensity and a 43% reduction in median flush duration relative to an older formulation of NER (59). In NER clinical trials, not more than 6% of subjects discontinued because of flushing (60). Flushing side effects are less with 1000 mg than 2000 mg of NER, and still retain roughly 70% of the improvement in HDL-C. Once daily dosing of NER in a 59 week-study showed only two subjects out of 723 having aspartate aminotransferase (AST) levels > 3 times the upper limit of normal (ULN) and no subjects with alanine aminotransferase (ALT) > 3 times ULN (1). Trials involving the combination of NER and a statin also show little evidence of hepatotoxicity (61–63).

NER can be safely combined with statins to lower LDL-C, triglycerides and non-HDL-C, while raising HDL-C. This combination therapy does not appear to worsen flushing side effects, and NER has not been shown to potentiate statin-induced myopathies. Combination treatment with NER and simvastatin (NER/S, SIMCOR®, Abbott Laboratories) in recent clinical trials showed this dual therapy was well tolerated (64–66). In a randomised study, comparing combination NER/S to simvastatin alone (SEACOAST I), only 7.5% of subjects receiving 1000/20 mg/day or 2000/20 mg/day NER/S discontinued because of flushing (64). In a similar study (SEACOAST II), discontinuations caused by flushing were not significantly different in subjects receiving NER/S 1000/40 mg/day (4.3%) or 2000/40 mg/day (5.0%) relative to subjects receiving simvastatin monotherapy and 50 mg/day of IR niacin. In a long-term open-label study in subjects with dyslipidaemia (OCEANS) previously treated with simvastatin, the discontinuation rate of subjects receiving 2000/40 mg/day NER/S was only 7% because of flushing (66). As tolerance developed to niacin alone, tolerance similarly developed to NER/S, as > 60% of the subjects who flushed during the first 12-week titration phase did not flush during weeks 41–52 (66).

## Non-steroidal anti-inflammatory drugs

NSAIDs are convenient cotherapies with niacin to reduce flushing. They decrease the production of multiple prostaglandins by preventing COX from metabolising arachidonic acid. In a flush-provocative study, using healthy volunteers, aspirin given 30 min before a 2000 mg dose of NER decreased the incidence, duration and severity of flushing compared with placebo pretreatment (67). Among subjects receiving placebo, 77% of subjects reported flushing with newer formulations of ER niacin. However, only 53% of subjects receiving aspirin 30 min before a dose of a newer formulation of ER niacin flushed (67). Aspirin given concomitantly with NER was also effective at reducing flushing incidence, duration and severity compared with placebo, but not as effective as aspirin 30-min pretreatment (67). In a prospective, randomised, double-blind, placebo-controlled trial, 325 mg aspirin given 30 min before a dose of NER reduced both the number and intensity of flushing episodes, resulting in a lower rate of discontinuation because of flushing in the aspirin group compared with placebo (1.8% vs. 9.4%; p = 0.007) (68). Along with aspirin, indomethacin (33), ibuprofen (69) and naproxen (32) have been shown in subjects to decrease the flushing effects of IR niacin. The most common dose of aspirin used to effectively reduce flushing in IR niacin is 325 mg, with 650 mg offering no further benefit (70). Aspirin use appears to have no negative impact on niacin’s decrease of free fatty acids (33).

## DP_1_ receptor antagonists

Recent efforts to decrease the flushing effects of niacin have focused on eliminating the downstream effects of prostaglandins that play a role in cutaneous vasodilatation. A highly selective DP_1_ antagonist, laropiprant [LRP, Merck/Merck Sharp & Dohm (MSD)] is currently in clinical trials to be used concomitantly with an alternate formulation of extended-release niacin from NER. In a small clinical trial, using healthy volunteers, LRP administered along with niacin decreased the symptoms of flushing compared with niacin plus placebo (71). However, about 70% of subjects taking a clinically relevant dose (30 mg) of LRP along with 1500 mg of niacin still flushed (71). This high incidence is likely caused by other pathways involved in the niacin flushing response. LRP is highly selective for DP_1_ and has no affinity for inhibiting the EP_2_ or EP_4_ receptors (72), which are also highly likely involved in flushing (12). This situation underscores the difficulty in modulating the downstream actions of prostaglandins for pharmacological effect.

## GPR109 antagonists and other agents

Inhibiting the GPR109 receptor would theoretically mitigate the flushing response. Antagonism of the GPR109 receptor to reduce flushing while preserving adipocyte antilipolytic activity would require targeting the Langerhans-specific GPR109A receptor. The potential side effects are unknown. Another potential target would be to inhibit the ability of PLA_2_ to produce arachidonic acid, thereby eliminating the production of prostaglandins upstream of COX. Glucocorticoids can indirectly inhibit PLA_2_ (73,74), but there are currently no approved therapies that specifically target this enzyme.

## Patient education

One of the easiest ways that medical personnel can help improve their patients compliance with niacin therapy is to provide their patients with a clear understanding of the clinical benefits of niacin as well as what to expect and how to manage flushing (48). While improvements in lipid profiles are meaningful to physicians, patients may be more willing to tolerate the transient flushing symptoms that can occur if they realise that niacin reduces cardiovascular risk, as determined by both mortality and cardiovascular event rates, and that this benefit extends beyond the duration of active therapy. Patients should be counselled to take aspirin (325 mg) 30 min before a snack and extended-release niacin. The importance of continuing to take the final daily maintenance dose of niacin extended-release should be emphasised. If there is a period of discontinuation, the titration procedure has to be followed again, although it maybe possible to accelerate it at the discretion of the prescriber. Patients are also advised to avoid hot beverages, spicy foods and hot showers near the time of taking niacin.

## Conclusions

Given niacin’s beneficial effects across a wide spectrum of the lipid profile, proven safety record and ability to be safely combined with statins, it should be regarded as an attractive option for the treatment of dyslipidaemia. The same receptor that is responsible for niacin’s decrease in free fatty acids is also likely the same receptor that is responsible for the flushing side effects. Therefore, it is currently difficult to separate the two effects, but flushing can be effectively managed in patients. As tolerance to flushing develops rapidly, healthcare professionals should particularly address flushing during dose titration of niacin. Clinically, unlike LRP, aspirin is not only an established agent to reduce flushing but it is also indicated for use in most dyslipidemic patients to reduce atherothrombotic complications, which is also the reason to prescribe niacin. As COX is upstream of PGD_2_, NSAIDs have the ability to block production of PGD_2_, PGE_2_ and PGI_2_. LRP has only the ability to block PGD_2_-mediated flushing. Initial data indicate that aspirin or LRP combined with extended-release niacin formulations have similar impacts on niacin-induced flushing in patients, although it is difficult to directly compare separate clinical studies. A clinical trial to assess the relative efficacy of these two agents is needed. At least in mice, deletion of the COX enzyme decreased flushing by almost 100%, while elimination of the DP_1_ receptor only decreased flushing by 40% after niacin treatment. Aspirin has a well-known safety profile, while long-term data on the safety of LRP is awaited. Future therapies that can preserve or even enhance niacin’s important lipid effects while eliminating flushing will likely be important improvements in the treatment of dyslipidaemia.
